# Genetic and Biological Characteristics of Gruid Herpesvirus 1 Isolated From Wild Cranes Affected by Inclusion Body Disease of Cranes

**DOI:** 10.1155/tbed/2658800

**Published:** 2025-05-24

**Authors:** Kemi Ishikawa, Hitoshi Hatai, Masayuki Horie, Makoto Ozawa, Yukiko Tomioka, Moe Ijiri, Kaori Tokorozaki, Tomohito Ikeda, Satoshi Maruyama, Mana Esaki, Yoshikazu Fujimoto

**Affiliations:** ^1^Joint Faculty of Veterinary Medicine, Kagoshima University, 1-21-24 Korimoto, Kagoshima 890-0065, Japan; ^2^Farm Animal Clinical Skills and Disease Control Center, Faculty of Agriculture, Iwate University, 3-18-8 Ueda, Morioka, Iwate 020–8550, Japan; ^3^Graduate School of Veterinary Science, Osaka Metropolitan University, Izumisano Osaka 598-8531, Japan; ^4^Osaka International Research Center for Infectious Diseases, Osaka Metropolitan University, Osaka 545-0051, Japan; ^5^Kagoshima Crane Conservation Committee, Izumi Kagoshima 899-0208, Japan; ^6^Laboratory of Laboratory Animal Science, Joint Department of Veterinary Medicine, Faculty of Agriculture, Tottori University, Tottori 680-8553, Japan

## Abstract

Inclusion body disease of cranes (IBDCs) is fatal in many cases and reportedly caused by a herpes-like virus labeled as gruid herpesvirus 1 (GrHV-1). Although GrHV-1 has been isolated from IBDC-affected cranes, it has not been genetically classified because its genome has not been partially or fully sequenced. In this study, we isolated an alphaherpesvirus from hooded cranes (*Grus monacha*) diagnosed with IBDC in Japan. Next-generation sequencing revealed that this virus isolate was GrHV-1, based on the 99.98% sequence homology with a previously isolated GrHV-1 strain. Furthermore, phylogenetic analysis of eight conserved herpesvirus genes supported the taxonomic assignment of GrHV-1 to the genus *Mardivirus* of the Alphaherpesvirinae subfamily. Based on these results, GrHV-1 can be more accurately classified and diagnostic tools to investigate suspected cases of IBDC can be developed. Furthermore, GrHV-1 showed effective replication in primary cultured cells derived from duck and chicken embryos and embryo tissues, highlighting the importance of further studies to evaluate its interspecies transmission.

## 1. Introduction

Inclusion body disease of cranes (IBDCs) is a fatal viral disease and its causative pathogen is referred to as gruid herpesvirus 1 (GrHV-1). IBDC was reported in cranes of different species in zoos and safari parks in Austria, USA, France, and Japan between 1979 and 1997 [1–4]. IBDC cases show nodular lesions in the liver and spleen grossly and can be diagnosed based on the characteristic microscopic inclusions in cell nuclei throughout the histopathological lesions. Virus has been successfully isolated from lesions in several cases and shown to have herpesvirus-like features physicochemically and morphologically [2, 5], giving rise to the GrHV-1 nomenclature [3, 5, 6]. However, to the authors' knowledge, the GrHV-1 genome has not previously been sequenced, and GrHV-1 is regarded by the International Committee on Taxonomy of Viruses as an unassigned virus in the family Herpesviridae [7]. Furthermore, no further reports of IBDC have appeared in the literature this century. Thus, there is a need for further research on the epidemiology of this virus and its molecular characterization.

The emergence of suspected IBDC cases at an overwintering site for migratory cranes in southern Kyushu, Japan, provided an opportunity for further research on GrHV-1. This overwintering site is one of the largest of its kind in Japan and draws a wide variety of wild birds who migrate between Alaska, the Russian Far East, China, and other East Asian regions. Approximately 90% of the world's population of hooded cranes (*Grus monacha*) and half of the world's population of white-naped cranes (*Grus vipio*) spend the winter at this site and both these species are categorized as vulnerable on the International Union for Conservation of Nature Red List [8]. Birds at this overwintering site are subject to epidemiological surveillance, mainly targeting avian influenza; however, in the 2018 winter season, we observed symptoms and gross lesions in hooded cranes that had become debilitated and we suspected these cranes had developed IBDC. Accordingly, we aimed to isolate virus from pathological tissue in these birds and investigate the biological and genomic characteristics of the viral isolates. Furthermore, we sequenced a previously isolated GrHV-1 strain (Hino93-1, isolated from a crane with IBDC in a Japanese zoo in 1993) [5] for comparative phylogenetic analyses with our 2018 viral isolates from the overwintering site.

## 2. Materials and Methods

### 2.1. Pathological Examinations

Cranes that died after showing debilitation at an overwintering site (in southern Kyushu, Japan) between October 2018 and March 2019 were necropsied and submitted for histopathological examinations in cases where IBDC was suspected. Tissues were collected from the deceased cranes, fixed with 10% formalin neutral buffer solution (Wako), and embedded in paraffin wax. Sections were dissected at a thickness of 3 μm and stained with hematoxylin and eosin (HE).

### 2.2. Cells

Primary chicken embryo fibroblasts (CEFs) and duck embryo fibroblasts (DEFs) were prepared from 10-day-old white leghorn and 11-day-old Pekin duck embryos. Chicken liver hepatocellular carcinoma (LMH) and chicken fibroblast (DF-1) cell lines were purchased from the Japanese Cancer Research Resources Bank (JCRB) Cell Bank and the American Type Culture Collection (ATCC), respectively. Vero cells from the stock maintained in our laboratory were used in this study. All cell lines were cultured in Dulbecco's modified eagle's medium (DMEM; Gibco) containing 10% fetal bovine serum at 37°C in an atmosphere containing 5% CO_2_.

### 2.3. Virus Isolation

To isolate viruses, we collected swab samples and organs from the deceased cranes identified at the overwintering site. Conjunctival, oropharyngeal, and cloacal swabs were collected in a 3-mL viral transport medium (Becton Dickinson and Company). We collected the liver, spleen, esophagus, rectum, and kidneys and prepared a homogenate for each organ in 10% *w*/*v* DMEM. Each homogenate was centrifuged at 2200 × *g* for 5 min to obtain the supernatant for analysis. One hundred microliters of each collected supernatant was inoculated into DEFs cultured on six-well culture plates and the plates were incubated for 5 days. The cells were harvested after three freeze–thaw cycles and three blind passages were performed until observation for cytopathic effects (CPEs). After centrifugation, the supernatant was stored at −80°C until use for analysis.

The Hino93-1 strain had been isolated from the liver of a red-crowned crane (*Grus japonensis*) diagnosed with IBDC at a zoo located in Tokyo, Japan, in 1993 [5]. The Hino93-1 virus used in this study was a gift from Hiroshi Suzuki (Tokyo Metropolitan Livestock Hygiene Service Center) and was propagated on DEFs.

### 2.4. Electron Microscopy

DEFs were inoculated with GrHV-1 and incubated until a CPE was observed in significant number of the cells. Cultures were collected and centrifuged at 10,000 × *g* for 30 min to remove cell debris and the supernatant was collected. For pelleting of virus, 25 mL of the supernatant was layered on top of a 10 mL 20% (*w*/*v*) sucrose cushion in phosphate-buffered saline (PBS) and centrifuged for 2 h at 175,000 × *g* at 4°C using an Optima XE-90 (Beckman Coulter, Inc.). The supernatant was discarded and the pellet was resuspended in 1 mL of PBS.

Virus particles stained with 2% phosphotungstic acid (pH 6.8) were observed using a transmission electron microscope (JEM-1400Plus, JEOL) at 80 kV.

### 2.5. Identification of Herpesvirus Genome

Nucleic acids were extracted from cell culture supernatants and swabs collected from the deceased cranes using the innuPREP Virus DNA/RNA Kit (Analytic Jena). Subsequently, a polymerase chain reaction (PCR) was performed using Takara ExTaq DNA Polymerase (Takara Bio) with extracted nucleic acid as a template and consensus primer sets for the herpesvirus polymerase gene [9]. PCR products were analyzed on a 1.5% agarose gel made up in tris-borate EDTA buffer containing 0.5 μg of ethidium bromide per mL and then a specific fragment of cDNA was purified using the Monarch DNA Gel Extraction Kit (New England Biolabs). To determine the sequence of the amplified DNA, sequencing analysis was conducted using a BigDye Terminator v3.1 cycle sequencing kit and an ABI 3130 sequencer (Applied Biosystems).

### 2.6. Complete Genome Sequencing

Nucleic acids were extracted from GrHV-1 propagated on DEFs using the InnuPREP Virus DNA/RNA Kit. DNA libraries were prepared using a NEBNext Ultra II FS DNA Library Prep Kit (Illumina) and next-generation sequencing (2 × 150 bp) was performed using NextSeq 500 (Illumina). The obtained reads were preprocessed by fastp v0.20.0 [10] and assembled by metaSPAdes 3.12.0 [11]. Assembled contigs with a length exceeding 1000 nucleotides were extracted using SeqKit v0.9.0 [12] and alphaherpesvirus contigs were identified using BLASTx [13]. Putative open reading frames (ORFs) on the viral genomes were searched for using EMBOSS getorf [14]. The genome sequences established in this study have been deposited in the DDBJ database under accession numbers LC810221 (Strain 5591Scl) and LC810222 (Strain Hino93-1).

### 2.7. Phylogenetic Analysis

A molecular phylogenetic tree was constructed using the maximum likelihood method in MEGA X version 10.1.7. The reliability of the tree was assessed by bootstrap resampling (*n* = 1000).

### 2.8. Growth Kinetics of GrHV-1 in Cultured Cells

To investigate the viral growth kinetics in cultured cells including CEFs, DEFs, LMH, DF-1, and Vero cells, we seeded cells onto a six-well plate (5 × 10^5^ cells per well). Each virus was absorbed onto the cells at a multiplicity of infection (MOI) of 0.005 for 1 h and the cells were then washed to remove nonadherent virus and incubated in 1.5 mL of DMEM at 37°C. At 24-h intervals, the supernatant was harvested from three independent wells. The virus titer of the supernatant was determined for DEFs seeded onto a 48-well-plate at a median tissue-culture infectious dose (TCID_50_).

### 2.9. Pathogenicity of GrHV-1 in Chicken and Duck Embryos

Two hundred microliters of each virus solution adjusted to 10^4^ TCID_50_ was inoculated into the allantoic cavity or yolk sac of an embryonated egg, which was then incubated at 37°C. Eleven-day-old chicken embryonated eggs and 13-day-old duck embryonated eggs were used for viral inoculation via the allantoic cavity route, and 7-day-old chicken embryonated eggs and 10-day-old old duck embryonated eggs were used for viral inoculation via the yolk sac route. The eggs were observed daily for 10 days for survival or mortality and the day of embryonic death was recorded in each experiment. The Japanese laws on animal research state that experiments with fertilized eggs performed before hatching do not require animal ethics approval.

## 3. Results

### 3.1. Pathological Examination

**Figure 1:**
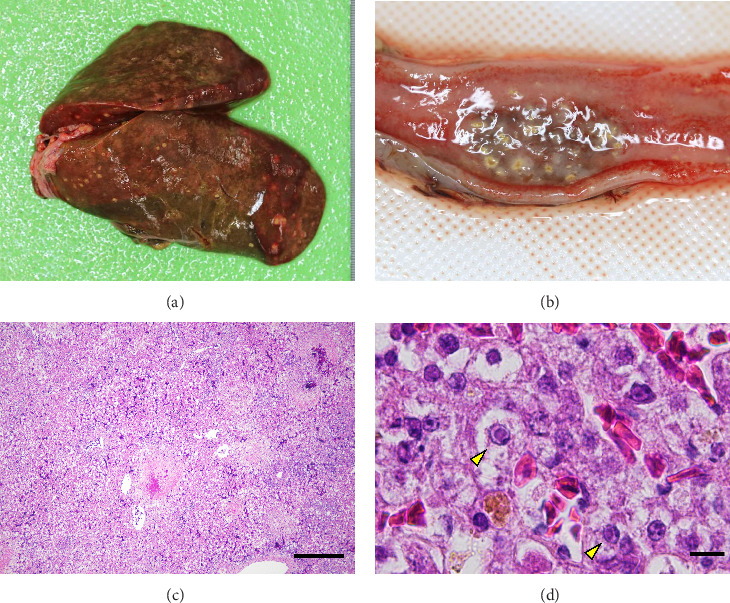
Gross pathological and histopathological findings in hooded cranes that died following debilitation. Yellowish-white nodules were observed in the liver (A) and the rectum (B). (C) Multiple necrotic foci were observed in the liver. Scale bar = 100 μm. (D) Cowdry A-type inclusion bodies were observed in the nuclei of the hepatocytes (arrowheads). Scale bar = 10 μm.

A total of 41 cranes that died between October 2018 and March 2019 were necropsied and IBDC was suspected in four of these cases based on multiple small-sized (1–3 mm in diameter) yellowish-white nodules macroscopically observed in the liver, spleen, kidneys, and gastrointestinal tract (oral mucosa, pharynx, esophagus, small intestine, and rectum; Figure 1A,B). Furthermore, these cases also showed congestion in the liver, follicular hypertrophy in the spleen, pseudomembranes in the esophageal mucosa, and multiple petechiae in the cecal tonsil and bursa of Fabricius. The histopathological correlates encompassed coagulation necrosis (Figure 1C) and Cowdry A-type or full-type intranuclear inclusions with nuclear membrane thickening in hepatocytes and Kupffer cells around the necrotic foci (Figure 1D), and these findings were the basis for a diagnosis of IBDC.

### 3.2. Virus Isolation From Cranes Diagnosed With IBDC

To isolate virus from cranes histopathologically diagnosed with IBDC, we processed tissues homogenates from the trachea, liver, spleen, lower rectum, and kidney of each bird for viral isolation, as described in the Methods section. Obtained tissue homogenate supernatants were inoculated into DEFs to isolate virus, in the conventional method previously described by other research groups [2, 5, 15]. A CPE was observed with cell shrinkage and syncytium formation in cells inoculated with samples from each of four deceased cranes (IDs C5591, C5592, C5593, and C19034; Table 1 and Figure S1). We then extracted DNA from the supernatants and subjected it to PCR analysis targeting partial DNA of the herpesvirus polymerase gene, as described in a previous report [9]. PCR products of the expected size were amplified from each isolate supernatant (data not shown). The nucleotide sequences of the obtained PCR products were determined and subject to a BLAST search. Although no significant similarity to database sequences of nucleotide was identified in this search, the predicted amino acid sequence showed the highest homology (72.3%) with the DNA polymerase protein sequence (UL30) of *Spheniscid alphaherpesvirus 1* (SpAHV-1; YP_009342373.1).

Virus isolated from the spleen of one deceased crane (ID C5591) was designated as strain 5591S and subjected to three rounds of limiting dilution cloning in DEFs to obtain pure virus (5591Scl) for further analysis. Electron microscopic observation of the 5591Scl pure virus strain revealed that its virions were spherical structures with a particle size of 100–150 nm and that the virion surfaces were covered with spiked projections (Figure S2). Morphologically, the 5591Scl strain closely resembled a GrHV-1 strain, Hino93-1, that had been previously isolated from a red-crowned crane diagnosed with IBDC [5]. Furthermore, the 5591Scl and Hino93-1 strains had an identical nucleotide sequence for the partial DNA polymerase gene. These results indicate that the virus isolated from the deceased cranes in this study was GrHV-1.

### 3.3. Detection of GrHV-1 Genes From Swab Samples Collected From Wild Cranes

To investigate the shedding of GrHV-1 from cranes, we submitted conjunctival, oropharyngeal, and cloacal swabs collected from deceased or debilitated birds for PCR analyses targeting viral polymerase genes, as described above. The swab specimens were collected from 287 hooded cranes, 53 white-naped cranes, and three sandhill cranes (*Grus canadensis*) between the 2016/2017 and 2021/2022 winter seasons. Viral genes were detected in samples from 37 hooded cranes (positive rate: 12.9%) and two sandhill cranes, though not in white-naped cranes. All amplified band sequences were identical to the target sequence of the 5591Scl strain. The PCR positive-cranes showed viral gene detection rates of 81.8% (27 positive/33 samples) for conjunctival swabs, 94.6% (35 positive/37 samples) for oropharyngeal swabs, and 81.3% (26 positive/32 samples) for cloacal swabs. In addition, viral growth was detected in DEFs, in several PCR-positive swabs, with viral titers of up to 2.8 log TCID_50_ ml^−1^.

### 3.4. GrHV-1 Genome Sequencing

**Figure 2:**
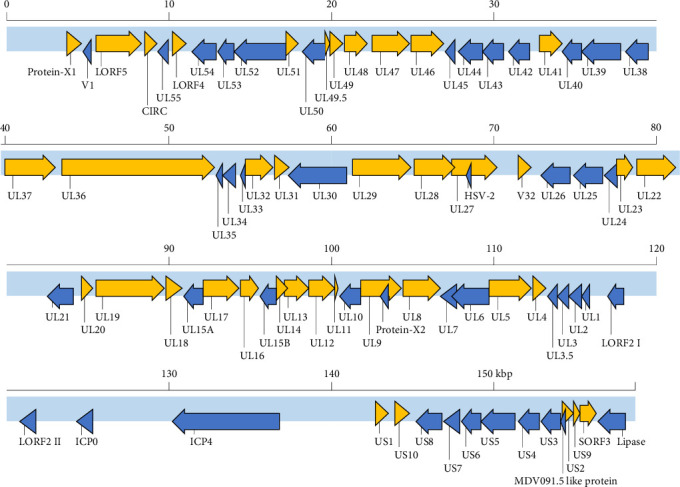
Schematic of the genome structure of GrHV-1. ORFs highlighted in yellow and blue were encoded on the positive strand and the negative strand, respectively.

**Figure 3:**
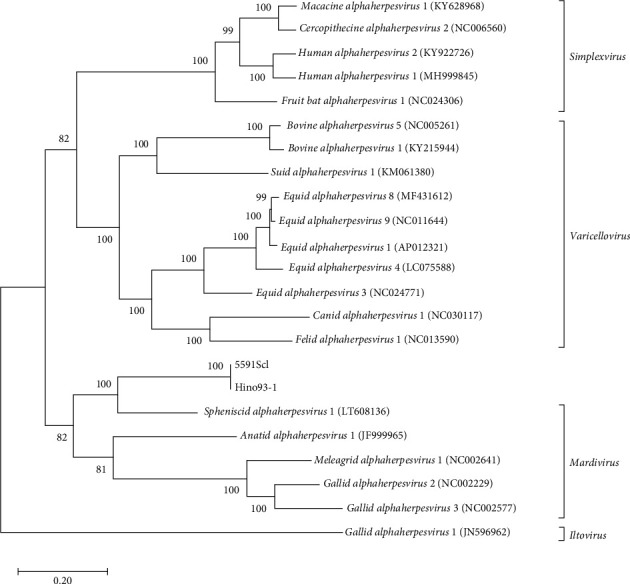
Phylogenetic relationship of GrHV-1 to other alphaherpesviruses. The phylogenetic tree was constructed from the amino acid sequences of eight ubiquitous genes (UL2, UL5, UL15, UL19, UL27, UL28, UL29, and UL30) of mammalian and avian alphaherpesviruses. The bootstrap values are shown for each interior branch.

The identification of GrHV-1 has relied on comprehensive evaluations covering the clinical condition of possibly infected cranes and the morphological and biochemical characteristics of viruses isolated from them and there is a total paucity of published genome sequence data for GrHV-1 required to allow virus identification in molecular analyses. Accordingly, to determine the complete genome sequence of GrHV-1, we submitted DNA samples extracted from the 5591Scl and Hino93-1 strains for next-generation sequencing. We found 99.98% identity between the complete 5591Scl and Hino93-1 genome sequences. Each of two viral genomes had a total length of approximately 159 kbp and a G + C content of 38.85%. A total number of 83 ORFs were predicted in each genome using EMBOSS getorf (Figure 2). The gene constellations were consistent across the two strains. Phylogenetic analysis based on the amino acid sequences of eight ubiquitous genes (UL2, UL5, UL15, UL19, UL27, UL28, UL29, and UL30) of mammalian and avian alphaherpesviruses [16] revealed that both the 5591Scl and Hino93-1 strains are most closely related to viruses of the genus *Mardivirus*. GrHV-1 is the most distant member within this group and its closest relative is SpAHV-1 (Figure 3).

### 3.5. Viral Growth Kinetics on Cultured Cell Lines

**Figure 4:**
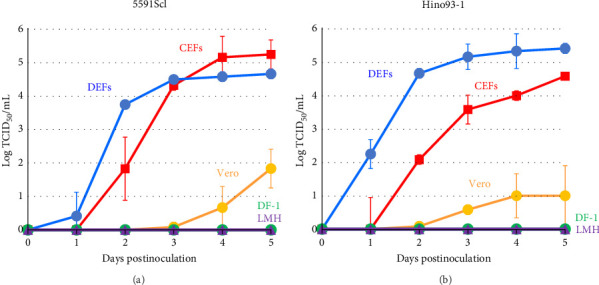
Growth kinetics of GrHV-1 in cultured cells. GrHV-1 strains 5591Scl (A) and Hino93-1 (B) were inoculated onto DEFs, CEFs, Vero, DF-1, and LMH cells at MOI of 0.005. The supernatant was collected at 1, 2, 3, 4, and 5 days post-inoculation. The virus titer of the supernatant was measured in DEFs by TCID_50_ assay.

The growth kinetics of GrHV-1 have not previously been investigated in avian-derived cell lines, although DEFs have previously been used in the conventional method for isolating herpes-like viruses from cranes diagnosed with IBDC. As shown in Figure 4, in both GrHV-1 strains investigated in this study (the 5591Scl and Hino93-1 strains), the titer in DEFs reached approximately 5.0 log TCID_50_ ml^−1^ by 2–3 days postinfection. We investigated the growth potential of GrHV-1 in chicken-derived, CEF, DF-1, and LMH cell lines (Figure 4). Both GrHV-1 strains showed slower growth in CEFs than in DEFs; however, their final yields were almost the same in both cell lines. DF-1 cells, which are immortalized CEF cells, did not support the growth of either the 5591Scl or Hino93-1 strain. Virus replication was not observed in chicken liver-derived LMH cells through 5 days postinfection. We also measured the viral titer in Vero cells derived from African green monkey kidneys, which are commonly used to isolate alphaherpesviruses. Both GrHV-1 strains were able to replicate in Vero cells; however, their titers were 100- to 1000-fold lower than those in DEFs and CEFs at 5 days postinfection (Figure 4).

### 3.6. Pathogenicity of GrHV-1 in Duck and Chicken Embryos

There is a paucity of data on the pathogenicity of GrHV-1 in poultry and the susceptibility of these birds to the virus. Thus, to investigate the pathogenic potential of GrHV-1 in poultry, we inoculated duck or chicken embryos with the 5591Scl or Hino93-1 strain via the yolk sac or allantoic cavity and monitored embryo survival versus mortality. The results are summarized in Table 2. Following inoculation with 5591Scl by the yolk sac route, duck and chicken embryos showed respective mortality rates of 75% and 90%, and respective mean times to death (MDT) of 3.1 and 5.1 days. Following inoculation with Hino93-1 by the yolk sac route, duck and chicken embryos showed respective mortality rates of 83% and 100%, and respective MDTs of 4.3 and 5.1 days. Almost all cases of duck or chicken embryonic death showed gross lesions encompassing stunted growth, systemic hemorrhage, pericardial effusion, and hepatic necrosis. We recovered virus from the brains and livers in cases of embryonic death and found titers ranging from 3.2 to 5.7 log TCID_50_/g. Following inoculation with 5591Scl by the allantoic cavity route, duck embryos showed a mortality rate of 75% and an MDT of 6.6 days; however, all chicken embryos survived through the end of the observation period. Following inoculation with Hino93-1 by the allantoic cavity route, duck embryos showed a mortality rate of 75% and an MDT of 5.9 days; by contrast, chicken embryos showed a mortality rate of 17% (embryonic death in one of six cases evaluated). These results suggest that chickens and ducks are susceptible to GrHV-1 infection, although duck embryos were more severely affected than chicken embryos. Additionally, we found a similar trend in pathogenicity and viral replication between duck and chicken embryos following infection with 5591Scl and Hino93-1.

## 4. Discussion

In this study, we investigated virus isolated from deceased cranes suspected to be IBDC. To the authors' knowledge, this is the first report on the genomic sequence of the causative virus of IBDC and GrHV-1, presenting crucial findings on the identification and pathogenicity of this virus. We successfully isolated virus from four hooded cranes histopathologically diagnosed with IBDC and found identical nucleotide sequences for our isolates from four cranes (IDs C5591, C5592, C5593, and C19034) and a previously isolated GrHV-1 strain (Hino93-1) in PCR analysis targeting the alphaherpesvirus polymerase gene. A representative strain isolated from one of the deceased cranes (Strain 5591Scl) was then subjected to next-generation sequencing, which revealed that its full genome sequence closely resembled that of a GrHV-1 strain (Hino93-1) isolated from a crane that died with IBDC at a Japanese zoo in 1997. These results demonstrated that the virus isolated in this study was GrHV-1 and indicate that GrHV-1 is an alphaherpesvirus. Our findings represent a major advance in the understanding of the viral pathogen responsible for IBDC, for which a betaherpesviruses classification had previously been considered possible, based on tentative physicochemical and morphological findings for virus isolated from cranes with IBDC in 1989 [3]. Irrespective of that report, diagnoses of IBCD up to the present have depended on the characteristic histopathological presence of multifocal necrosis and Cowdry type A-intranuclear inclusion bodies in the liver, spleen, gastrointestinal tract, and bursa of Fabricius, as reported in cranes naturally or experimentally affected with IBDC [1, 4, 17]. Hereafter, GrHV-1-induced IBDC may be more reliably diagnosed using genetic analysis, in addition to histopathological assessments.

We were able to throw some light on species-related differences in susceptibility to IBDC. Our survey of tissue samples from dead or debilitated birds in a number of species revealed GrHV-1 infection in 37 hooded cranes and two sandhill cranes, but not in white-naped cranes. Our findings are, thus, consistent with previous reports on differences between crane species in susceptibility and severity for this virus [5, 6, 18]. In previous outbreaks of IBDC, birds in eight crane species (including hooded and sandhill cranes) have reportedly shown high susceptibility to GrHV-1 infection (with signs of a herpes-like virus and/or detectable anti-virus antibodies), but white-naped cranes have not (no detectable virus antigen or anti-virus antibody reported). Interestingly, the crane species listed above may not be the only species susceptible to GrHV-1 infection. Individual birds in the Anatidae and Ciconiida families co-housed with cranes at a Japanese zoo reportedly showed seroconversion for a GrHV-1 strain (Hino93-1) during an historic IBDC outbreak [4]. Ducks have also been lethally infected with GrHV-1 [2, 19]. These reports suggest that GrHV-1 may spread to a diverse range of hosts (including noncrane species), especially in zoos, where different avian species are housed in close proximity. Further investigations of species susceptibility to GrHV-1 are, thus, necessary, together with further continuous epidemiological monitoring, both for cranes, who have been the main reported host of GrHV-1 so far, and other avian species.

Our study is also the first to provide any evidence on how IBDC is spread and GrHV-1 is shed by infected birds in the wild. We detected viral genes in PCR analysis of conjunctival, oropharyngeal, and cloacal swabs, and then, successfully isolated virus from swabs of each type, suggesting that cranes with IBDC shed GrHV-1 via a variety of excretory routes. The only previous attempt to isolate virus from wild birds with IBDC involved collection of cloacal swabs from approximately 70 cranes at a location that had seen crane die-offs in the late 1970s (the International Crane Foundation, Wisconsin, USA), but this attempt was not successful [18]. Thus, this study offers new evidence that IBDC is likely to spread by direct contact between infected birds and other susceptible birds and by contact with a virus-contaminated environment.

In phylogenetic analysis, we revealed that GrHV-1 is most closely related to SpAHV-1, which is classified as a member of the genus *Mardivirus* in the subfamily Alphaherpesviridae (Figure 3). We found several similarities between the GrHV-1 and SpAHV-1 viral genomes. These included the genes coding the V1 and V32 proteins, which have previously been observed only in varicelloviruses. Another example is the ICP0 gene, which is present in both the GrHV-1 and SpAHV-1 genomes, but absent from those of other avian herpesviruses of the genera *Mardivirus* and *Iltovirus* [20, 21]. Conversely, comparison of the genomes of these viruses based on the arrangement and organization of the putative protein revealed that the most important difference between the GrHV-1 and SpAHV-1 genomes is their structure. Herpesvirus genomes differ in the arrangement of direct and inverted repeat regions with respect to unique regions [22]. SpAHV-1 has a D-type genome that includes repeated sequences at one terminus and in an inverted orientation internally [20]. On the other hand, the GrHV-1 genome is classified as an F-type genome, in which the sequences at the two termini are not identical and are not repeated directly or are in an inverted orientation, resulting in a protein constellation between ICP4, which is located at approximately 130 kbp, and lipase, which is located at the genomic terminal (Figure 2); this constellation differs from that of SpAHV-1. These findings suggest that GrHV-1 and SpAHV-1 are descended from a common ancestral virus, from which they have evolved along independent paths. We tentatively named the two predicted ORFs in the GrHV-1 genome proteins X-1 and X-2, as they did not share significant sequence similarity with other herpesviral proteins. We anticipate future research on the mRNA expression of each ORF using RNA-seq to accurately investigate gene function in the replication of GrHV-1.

We also furnish evidence on the pathogenicity of GrHV-1 in poultry, which previously was unclear. The GrHV-1 isolate showed efficient growth in DEFs and infected duck embryo tissues, in which it was associated with high mortality (Figure 4 and Table 2). Our findings suggest that GrHV-1 is pathogenic in ducks and are consistent with reports of neurological symptoms in ducklings (age: 3–20 days) intramuscularly inoculated with the GrHV-1 strain Hino93-1 [19]. We also found that the GrHV-1 strains 5591Scl and Hino93-1 showed comparable growth in CEFs and DEFs (Figure 4) and chicken embryos inoculated with the 5591Scl strain via the yolk sac route showed high mortality and effective viral replication, although those inoculated by the amniotic cavity route did not (Table 2), indicating that GrHV-1 has the potential to replicate in chickens. Our findings present a contrast with those in a preliminary study where 16-day-old chickens (*n* = 2) survived intramuscular inoculation with a virus (possibly GrHV-1) isolated from an IBDC-associated crane [2]. We, thus, postulate that GrHV-1 may be pathogenic in both ducks and chickens, with greater pathogenicity in the former. Furthermore, Kaleta et al. [23] reported that quail herpesvirus (*Psittacid herpesvirus 1*: PsHV-1) isolated from dead bobwhite quails (*Colinus virginianus*) could cross-react with antiserum against the IBDC virus, probably GrHV-1, in a neutralization test. In addition, restriction endonuclease analysis showed that PsHV-1 and IBDC virus isolates have very similar restriction patterns [3, 23, 24]. No genetic information is available for the PsHV-1 genome. Genetic and biological similarities between GrHV-1 and PsHV-1 and the susceptibility of quails to GrHV-1 infection are of great interest.

Collectively, our data provide novel insights on GrHV-1, the viral pathogen of IBDC, and on viral diversity and evolution within the avian herpesvirus family. To the best of our knowledge, this is the first epidemiological study of GrHV-1 using molecular genetic techniques. Given that wild cranes can be captured and housed with other injured birds in protective facilities and that cranes are sometimes transferred to zoos (both within Japan and internationally) for breeding purposes, we recommend IBDC cases should be quarantined as infection with GrHV-1 can cause fatal diseases in cranes [6]. The results of the present study may, thus, be useful for the conservation of crane species.

## 5. Conclusion

IBDCs is a potentially fatal condition caused by GrHV-1. In this study, we determined the complete genome sequence of GrHV-1 isolated from hooded cranes affected by IBDC in Japan using next-generation sequencing. Phylogenetic analysis confirmed that the virus belongs to the genus *Mardivirus* of the Alphaherpesvirinae subfamily. This study is the first to report the genome sequence of GrHV-1, contributing to its accurate genetic classification. Additionally, this genomic information enables the development of new genetic diagnostic tools for IBDC, which has so far been diagnosed based on histological examination alone.

## Figures and Tables

**Table 1:** Isolation of GrHV-1 from tissues of cranes diagnosed with IBDC.

Crane ID	Species	Tissues samples					Isolated strain
		Liver	Spleen	Rectum	Esophagus	Kidney	
C5591	Hooded crane	+	+	+	+	NS	5591L, 5591S, 5591Rec, 5591ESO
	(*Grus monacha*)	2.8^a^	2.5	3.8	2.8		

C5592	Hooded crane	+	+	NS	NS	NS	5592L, 5592S
		3.8	3.8				

C5593	Hooded crane	+	+	−	NS	NS	5593L, 5593S
		4.5	3.3				

C19034	Hooded crane	+	+	−	NS	−	19034L, 19034S
		3.5	4.5				

*Note*: +, positive for virus isolation; −, negative for virus isolation.

Abbreviation: NS, no sample.

^a^Virus titer in tissues (log TCID_50_/g).

**Table 2:** Pathogenicity of GrHV-1 in duck and chicken embryos.

Inoculation route	Animal species	Virus strain	Mortality rate (%)	MDT (day)	Average virus titer in tissues of dead embryos (log TCID_50_/g ± SD)	
					Brain	Liver
Yolk sac	Duck	5591Scl	75	3.1	4.1 ± 1.5	3.2 ± 0.8
		Hino93-1	83	4.3	4.1 ± 1.2	4.0 ± 1.1
	Chicken	5591Scl	90	5.1	4.8 ± 0.7	5.7 ± 1.7
		Hino93-1	100	5.1	4.5 ± 0.9	4.5 ± 1.7

Allantoic cavity	Duck	5591Scl	75	6.6	NT	NT
		Hino93-1	75	5.9	NT	NT
	Chicken	5591Scl	0	—	—	—
		Hino93-1	17	6	NT	NT

Abbreviations: MDT, mean time of death; NT, not tested; SD, standard deviation.

## Data Availability

The data that support the findings of this study are openly available in DNA Data Bank of Japan at https://www.ddbj.nig.ac.jp/index.html, reference number LC810221 and LC810222.

## Supporting Information

Supporting Information Figure S1: Cytopathic effects in DEFs 2 days after inoculation with GrHV-1 strains. Figure S2: Electron microscopy image of a virus isolated from cranes diagnosed with IBDC.
