# Supplementary material for: Genetic and Biological Characteristics of Gruid Herpesvirus 1 Isolated From Wild Cranes Affected by Inclusion Body Disease of Cranes
Source: Transbound Emerg Dis. 2025 May 24;2025:2658800. doi: 10.1155/tbed/2658800 (PMC12126257; doi:10.1155/tbed/2658800)
Supplement: Supporting Information — Figure S1: Cytopathic effects in DEFs 2 days after inoculation with GrHV-1 strains. Figure S2: Electron microscopy image of a virus isolated from cranes diagnosed with IBDC. [file 2658800.f1.pptx]

## Slide 1
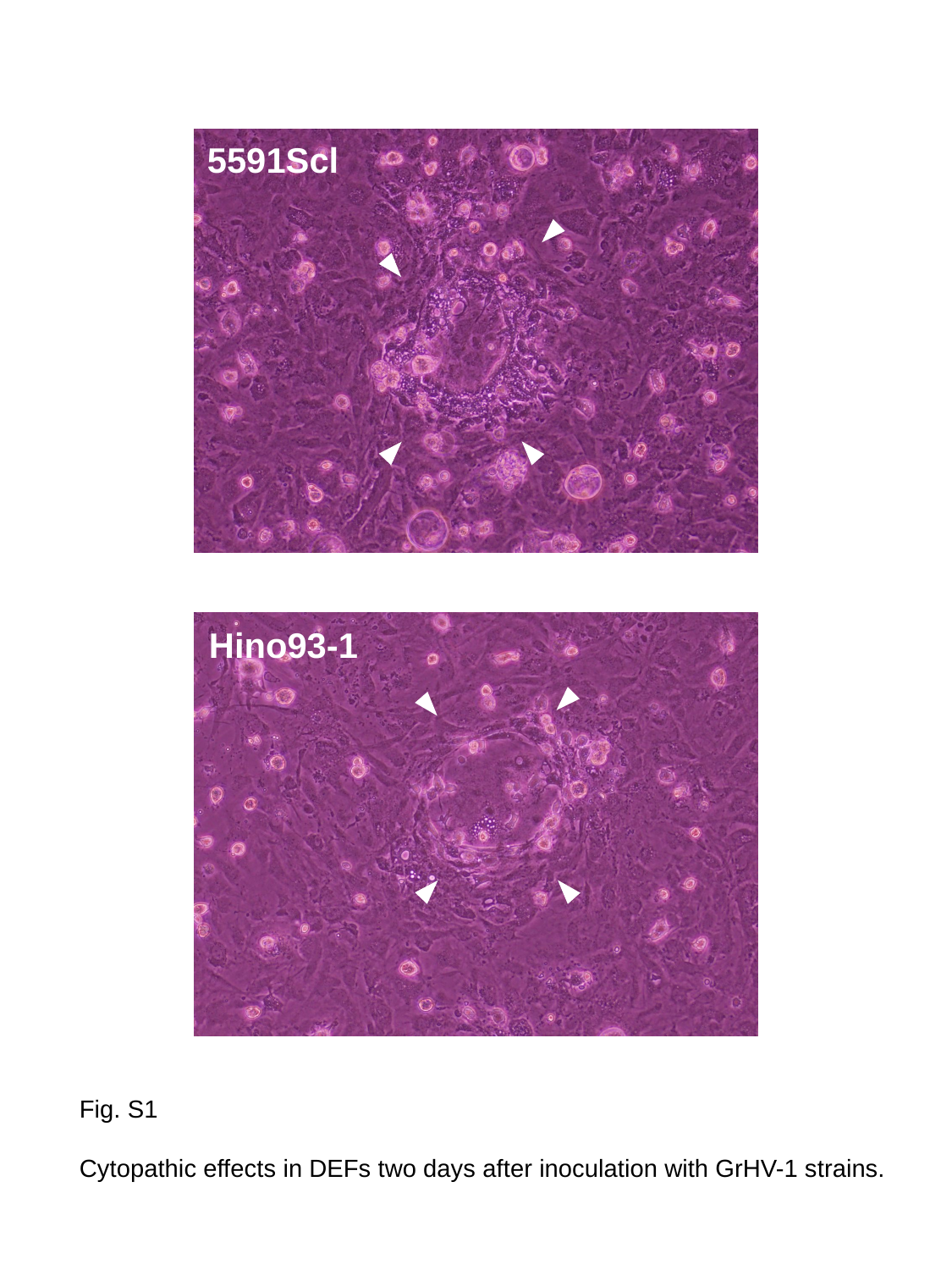

5591Scl
Hino93-1
Fig. S1
Cytopathic effects in DEFs two days after inoculation with GrHV-1 strains.

## Slide 2
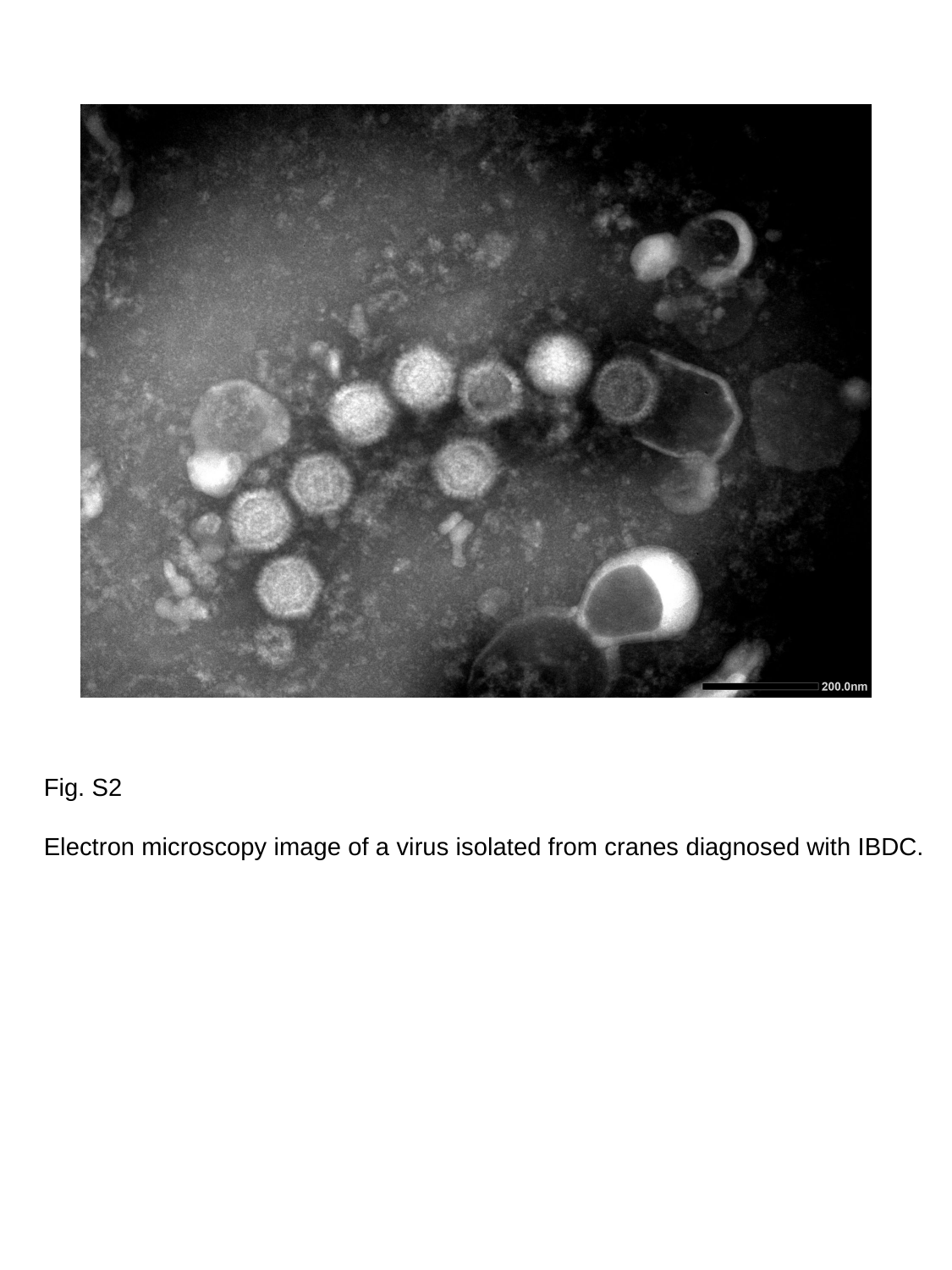

Fig. S2
Electron microscopy image of a virus isolated from cranes diagnosed with IBDC.
